# Circular RNA CircEYA3 induces energy production to promote pancreatic ductal adenocarcinoma progression through the miR-1294/c-Myc axis

**DOI:** 10.1186/s12943-021-01400-z

**Published:** 2021-08-21

**Authors:** Zeyin Rong, Si Shi, Zhen Tan, Jin Xu, Qingcai Meng, Jie Hua, Jiang Liu, Bo Zhang, Wei Wang, Xianjun Yu, Chen Liang

**Affiliations:** 1 Department of Pancreatic Surgery, Fudan University Shanghai Cancer Center, 270 Dong’An Road, Shanghai, 200032 PR China; 2 Department of Oncology, Shanghai Medical College, Fudan University, Shanghai, 200032 China; 3 Pancreatic Cancer Institute, Fudan University, Shanghai, 200032 China; 4 Shanghai Pancreatic Cancer Institute, Shanghai, 200032 China

**Keywords:** CircEYA3, miR-1294, C-Myc, ATP production, Pancreatic ductal adenocarcinoma

## Abstract

**Background:**

Extensive studies have demonstrated the pivotal roles of circular RNAs (circRNAs) in the occurrence and development of different human cancers. However, the expression and regulatory roles of circRNAs in pancreatic ductal adenocarcinoma (PDAC) are unclear.

**Methods:**

CircEYA3 was explored based on Gene Expression Omnibus (GEO) dataset analysis. qRT-PCR was applied to determine the expression of circRNAs, miRNAs and mRNAs in PDAC cells and tissues. The biological roles of circEYA3 in vitro and in vivo were determined by performing a series of functional experiments. Further, dual luciferase reporter, fluorescence in situ hybridization (FISH), RNA pull-down assays, and RNA immunoprecipitation (RIP) assays were used to confirm the interaction of circEYA3 with miR-1294.

**Results:**

CircEYA3 was elevated in PDAC tissues and cells, and a higher level of circEYA3 was significantly associated with a poorer prognosis in patients with PDAC. Functionally, circEYA3 increased energy production via ATP synthesis to promote PDAC progression in vitro and in vivo. Mechanistically, circEYA3 functions as an endogenous miR-1294 sponge to elevate c-Myc expression, thus exerting its oncogenic functions.

**Conclusion:**

CircEYA3 promotes the progression of PDAC through the miR-1294/c-Myc signalling axis, and circEYA3 may be an efficient molecular therapeutic target in PDAC.

**Supplementary Information:**

The online version contains supplementary material available at 10.1186/s12943-021-01400-z.

## Background

The prognosis of pancreatic ductal adenocarcinoma (PDAC), a highly aggressive and lethal malignancy, remains unsatisfactory, with a 5-year overall survival (OS) rate of less than 5% [1]. PDAC is going to have second-highest global rate of cancer-associated mortality within the next decade [2]. Despite the numerous clinical endeavours directed at PDAC patients, the outcome of PDAC remains unfavourable due to its low rate of early diagnosis and is characteristic of high metastasis [3]. Hence, determining the potential mechanism of PDAC tumorigenesis and increasing the efficiency of early PDAC diagnosis are urgently needed to develop novel therapeutic strategies for this cancer.

Circular RNAs (circRNAs), an abundant RNA species, are an enigmatic group of noncoding RNAs (ncRNAs) formed by unique back-splicing events; they have a continuous closed structure without a 5′ end or 3′ polyadenylated tail and have become a popular topic in RNA research [4, 5]. CircRNAs are acknowledged to be conserved across species and to be tissue- or developmental stage-specific, and they can resist exonuclease degradation [6]. In addition, circRNAs are translated and involved in the regulation of gene expression at both the transcriptional and translational levels [7]. Accumulating studies have shown that circRNAs are abnormally aberrantly expressed and participate in the pathogenesis of different cancers, such as lung cancer [8], gastric cancer [9], colorectal cancer [10], osteosarcoma [11], and melanoma [12]. In PDAC, some dysregulated circRNAs have been identified to play crucial roles in proliferation and progression. For example, according to previous reports, circBFAR acts as a miR-34b-5p sponge to facilitate the proliferation and metastasis of PDAC cells by activating MET signalling [13]. More recently, Shen et al. revealed that a negative feedback loop was formed between circNEIL3 and ADAR1 that can promote the progression of PDAC through the miR-432-5p/ADAR1/GLI1 axis [14]. To the best of our knowledge, although some dysregulated circRNAs have been reported to play crucial roles in the initiation and development of PDAC [15], here are still many unknowns that need exploration and study to elucidate the underlying mechanisms of circRNAs in PDAC.

In our present study, we focused on the upregulated circRNAs in PDAC based on GEO dataset analysis for the first time and characterized a novel oncogenic circRNA, circEYA3 (hsa_circ_0007895). CircEYA3 was derived from the EYA3 gene, exhibited an apparently elevated level in PDAC and was closely correlated with the prognosis of PDAC patients. Mechanistically, circEYA3 functioned as a sponge for has-miR-1294 (miR-1294) to elevate the level of c-Myc. c-Myc is highly important in PDAC [16], and circEYA3 was found to exert its oncogenic functions by elevating ATP production in PDAC cells. Collectively, our findings indicated that circEYA3 may be a novel prognostic biomarker and promising therapeutic target for PDAC.

## Materials and methods

### Tissue specimens and cell lines

A total of 104 PDAC and matched non-cancerous tissues were collected from patients who underwent surgical resection without preoperative chemotherapy at Fudan University Shanghai Cancer Center (FUSCC), between 2013 and 2017. Each sample was evaluated by two independent pathologists. This study was approved by the Clinical Research Ethics Committee of FUSCC. Written informed consent was acquired from each patient before participation in this study. Human PDAC cells (Capan-1, MiaPaCa-2, SW1990, PANC-1, BXPC-3, and CFPAC-1) and human pancreatic ductal epithelial (HPDE) cells were obtained from ATCC. Cells were cultured as previously mentioned [17, 18].

### Immunohistochemical (IHC) analysis

For IHC staining, slides were stained with antibodies against c-Myc (1:150, ab32072, Abcam), E-cadherin (1:400, #3195, CST), N-cadherin (1:50, #13116, CST), Bax (1:400, #14796, CST), cleaved caspase-3 (1:400, #9661, CST), and Ki-67 (1:400, #12202S, CST) following standard procedures previously described [17].

### RNA extraction, genomic DNA (gDNA) extraction, quantitative real-time PCR (qRT-PCR) analysis, RNase R treatment and actinomycin D (ActD) treatment

Total RNA was extracted from PDAC cells, tissues and matched non-cancerous tissues with TRIzol Reagent (Invitrogen, USA) in line with the manufacturer’s instructions. gDNA was isolated using a gDNA Reagent Kit (Sangon Biotech, Shanghai, China). cDNA was synthesized from 1 μg of total RNA using a PrimeScript RT Reagent Kit (Accurate Biology, China). qRT-PCR was performed with a SYBR Green PCR Kit (Accurate Biology, China) in an ABI 7900HT System (Applied Biosystems, USA) to determine the expression levels of candidate genes. The relative circRNA/mRNA and miRNA expression levels were normalized to those of GAPDH and U6, respectively, by using the 2 ^−△△CT^ method. For RNase R treatment, 10 μg of total RNA from the indicated cells was mixed with or without RNase R (3 U/μg) for 20 min at 37 °C. Next, circEYA3 and linear EYA3 mRNA were reverse transcribed with specific primers and analysed by qRT-PCR or PCR followed by nucleic acid agarose gel electrophoresis. For ActD treatment, cells were exposed to 2 μg/mL ActD (Sigma) at the indicated times. Then, the expression of the circRNA or linear mRNA was detected and analysed using qRT-PCR. Each experiment was carried out at least three times. All primers used in the experiment were synthesized by NovaBio (Shanghai, China) and are listed Additional file 1:Table S1.

### Oligonucleotides (oligos), plasmids and cell transfection

Small interfering RNAs (siRNAs) targeting circEYA3 and the relevant control siRNA were synthesized by RiboBio (Guangzhou, China). SiRNAs targeting c-Myc were synthesized by NovaBio (Shanghai, China). The sequences of the siRNAs are shown in Additional file 1: Table S2. To construct the circEYA3 overexpression plasmid, the full-length cDNA sequence of human circEYA3 by RiboBio (Guangzhou, China) and inserted into the pLCDH-circ expression vector to construct the circEYA3 overexpression vector pLCDH-circEYA3. The mimics, inhibitor and corresponding negative controls (NCs) for miR-1294 were synthesized by and purchased from GenePharma (Shanghai, China). The siRNAs, miRNA mimics and miRNA inhibitor were transiently transfected into cells with Lipofectamine 3000 (Invitrogen, CA, USA). To construct cell lines with stable overexpression of circEYA3, HEK-293 T cells were transfected with the circEYA3 overexpression vector pLCDH-circEYA3 and the control vector pLCDH-circ along with the psPAX2 packaging plasmid and pMD2.G envelope plasmid with Lipofectamine 3000. Forty-eight hours later, lentivirus was harvested and transduced into PDAC cells, which were then selected with 3 μg/mL puromycin (Sangon Biotech, Shanghai, China) for 2 weeks to establish the stable overexpression cell line.

### Cell proliferation assays

For the Cell Counting Kit-8 (CCK-8) assay, the treated PDAC cells were seeded into 96-well plates at a concentration of 3 × 10^3^/well. Then, 10 μL of CCK-8 assay solution (Dojindo, Japan) was added and incubated in the dark for 2 h. The absorbance at 450 nm was measured every 24 h with a microplate reader (BioTek Instruments, USA). For the EdU incorporation assay, an EdU cell proliferation assay kit (Beyotime, China) was used to detect the proliferation of treated PDAC cells. In brief, treated cells (100 μL of a 2 × 10^4^ cells/mL suspension) were seeded into 96-well plates, and 50 mM EdU was added at 37 °C for a 2-h incubation. After fixation with 4% paraformaldehyde and permeabilization with 1% Triton X-100, 100 μL of Click reaction cocktail was added to the treated cells, and the cells were then stained with Hoechst 33342. Immunostaining was imaged and quantitatively evaluated with a fluorescence microscope (Olympus, Japan). For the colony formation assay, equal numbers of treated PDAC cells were inoculated into 6-well plates and incubated at 37 °C for 2 weeks. At the end of the incubation period, the cells were fixed with 4% paraformaldehyde and stained with 1% crystal violet. Then, macroscopic colonies were photographed and counted. All experiments were repeated three times.

### Western blot analysis

In brief, proteins were isolated from PDAC cells and tumor tissues using RIPA buffer supplemented with proteinase and phosphatase inhibitors, and the protein concentration was determined with BCA reagent (Beyotime, China). Equal amounts of protein were electrophoresed on 10% SDS-PAGE gels and were then transferred onto PVDF membranes (Millipore), which were soaked for 2 h in 5% skim milk. Subsequently, the membranes were probed with primary antibodies specific for the following proteins: c-Myc (1:1000, ab32072, Abcam), E-cadherin (1:1000, #3195, CST), N-cadherin (1:1000, #13116, CST), Vimentin (1:1000, #5741, CST), Snail (1:1000, #3879, CST), Bax (1:1000, #14796, CST), cleaved caspase-3 (1:1000, #9661, CST), and β-actin (1:10000, 66,009–1-lg, Proteintech). Next, the membranes were incubated with the indicated secondary antibodies (1:1000, CST) for 1 h. After washing three times, the targeted proteins were visualized using enhanced chemiluminescence (ECL) reagent (Millipore, MA, USA). β-actin was used as the loading control in this study.

### Transwell migration and invasion assays

Transwell assays were conducted using 24-well plates with chamber inserts with 8.0 μm pores (Corning) without (migration) or with (invasion) 2% Matrigel (BD Science, USA). The cells were digested 24 h after transfection, and resuspended in serum-free medium. Then, 6 × 10^4^ cells per well were placed into the upper chambers. he lower chamber was filled with 500 μl medium with 10% FBS as a as a cell nutritional attractant. After incubation at 37 °C for 24 h, cells in the upper chamber were gently removed with a cotton swab. The migrated or invaded cells were fixed with 4% polyformaldehyde and visualized by staining with 0.2% crystal violet for 20 min. Last, the migrated or invaded cells were imaged and quantified by capturing five randomly chosen microscopic fields by using an inverted microscope (Olympus, Japan). All experiments were performed in triplicate.

### Three-dimensional (3D) spheroid invasion assays

3D spheroid invasion assays were carried out as previously reported [19]. The transfected cells were suspended with DMEM containing 10% FBS (2 × 10^4^ cells/mL), seeded into ultra-low attachment (ULA) round-bottom 96-well plates (200 μl/well), and cultured 4 days to form tumour spheroids. Next, 100 μl/well of DMEM from the spheroid plates was removed and then 100 μl of basement membrane matrix (BMM, Corning) was added into each well; the plates were centrifuged at 300×g for 3 min at 4 °C, and incubated at 37 °C to allow the BMM to solidify. One hour later, 100 μl DMEM containing 10% FBS was gently added into each well. Final, the spheroids were incubated at 37 °C in 5% CO2, and images were obtained using an inverted microscope (Olympus, Japan). All experiments were carried out in triplicate.

### Apoptosis assay

Apoptosis rates were measured with the Annexin V PE Apoptosis Detection Kit (BD) as reported previously [20]. In brief, the transfected cells were harvested and incubated with binding buffer supplemented with 5 μL of Annexin V-PE and 5 μL of 7-AAD for 15 min in the dark. Finally, apoptosis analysis was performed with a FACSCalibur flow cytometer. All experiments were carried out in triplicate.

### Fluorescence in situ hybridization (FISH) assay and PDAC TMAs

In brief, PANC-1 cells were grown in confocal dishes and fixed with 4% paraformaldehyde prior to permeabilization in PBS with 0.5% Triton X-100. The Cy3-labelled circEYA3 probes (Geneseed, Shanghai, China) and FAM-labelled miR-1294 probes (GenePharma, Shanghai, China) were diluted, denatured and added to PANC-1 cells at 37 °C overnight. The next day, cell nuclei were stained with DAPI for 15 min after hybridization. Finally, images were acquired with a Leica confocal microscope (Leica Microsystems, Germany). Moreover, the expression level of circEYA3 in tissues was evaluated by FISH in TMAs containing samples from 209 PDAC patients who were diagnosed with PDAC at the FUSCC, between 2014 to 2018. All samples were evaluated by two independent pathologists. This study was approved by the Clinical Research Ethics Committee of FUSCC. The intensity of circEYA3 staining was scored as follows: 0, no staining; 1, low staining; and 2, high staining. The percentage of positively stained cells was scored as follows: 0, 0% (no stained cells); 1, 1 ~ 24%; 2, 25 ~ 49%; 3, 50 ~ 74%; and 4, 75 ~ 100%. The final score was calculated by multiplying the scores for the staining intensity and the percentage of positively stained cells. Subsequently, the samples were divided into two groups: the low expression group (score 0–3) and the high expression group (score 4–8). The sequences of the circEYA3 and miR-1294 probes are listed in Additional file 1: TableS3.

### Dual-luciferase reporter assay

The full-length wild-type (WT) sequence of circEYA3, the c-Myc 3′ untranslated region (UTR) and the indicated mutant circEYA3 and c-Myc 3’UTR (Mut) containing the predicted miR-1294 binding sites were separately synthesized and cloned into the dual-luciferase reporter vector psi-CHECK-2 (Geneseed, Shanghai, China). The resulting dual-luciferase reporter plasmids (WT or Mut) were co-transfected with the miR-1294 mimic or inhibitor into PANC-1 or MiaPaCa-2 cells, respectively, using Lipofectamine 3000. After 48 h of incubation, the relative firefly luciferase activities with respect to the corresponding Renilla luciferase activities were measured and analysed using a Dual-Luciferase Assay System (Promega) following the manufacturer’s protocol.

### RNA pull-down assay

Pull-down assays with biotinylated circEYA3 were carried out as previously described [21]. In brief, a biotin-labelled probe specifically targeting circEYA3 and a random oligo probe (RiboBio, Guangzhou, China) were incubated with M280 streptavidin Dynabeads (Invitrogen, USA) at room temperature for 2 h. Lysates of PANC-1 and MiaPaCa-2 cells were incubated with the probe/bead complexes at 4 °C overnight. Subsequently, the circEYA3/miRNA/bead complexes were washed three times and eluted from the beads. Then, the enrichment of circEYA3 and related miRNAs in the precipitated complexes was evaluated by qRT-PCR. The sequences used in the pull-down assays are listed in Additional file 1: TableS4.

### RNA immunoprecipitation (RIP)

The RIP assay was conducted using a Magna RIP Kit (Millipore, Billerica, MA, USA) in accordance with the manufacturer’s instructions, as described previously [9]. In brief, an anti-Ago2 antibody or negative control immunoglobulin G (IgG; Millipore, USA) was applied for the RIP assay. Transfected cells were harvested and lysed in RNA lysis buffer and were then incubated with 5 μg of the anti-Ago2 antibody or IgG at 4 °C overnight. Then, 40 μL of magnetic beads were added. The next day, the RNA/bead complexes were washed and resuspended in Proteinase K buffer to separate proteins. Then, the immunoprecipitated RNA was purified and analysed by agarose gel electrophoresis or qRT-PCR. RIP assays were also performed in PANC-1 cells transiently overexpressing miR-1294 and the corresponding negative control.

### Measurement of ATP levels

ATP levels were measured as previously described using an ATP determination kit (Promega; FF2000) in accordance with the manufacturer’s instructions [22]. In brief, 1 × 10^6^ cells were mixed with 2.5% trichloroacetic acid (TCA) to extract ATP from the cell samples. After ATP extraction, the TCA in the sample was diluted to a final concentration of 0.1% by adding neutralizing Tris-acetate buffer (pH = 7.75). Then, 40 μL of the diluted sample was added to 100 μL of rL/L reagent (Promega; FF2000) for ATP measurement using a SpectraMax M5/M5e Multifunctional Microplate Detection System (Molecular Devices). The ATP standard was continuously diluted to obtain a standard curve for calculating the ATP concentration in each sample. The relative concentrations of ATP were calculated and normalized to those in the control groups. All experiments were performed in triplicate.

### Animal experiments

Animal studies were approved by the Committee on the Ethics of Animal Experiments of Fudan University. To establish xenograft mouse models, a lentiviral vector for stable knockdown of circEYA3 was designed based on the si-circEYA3–1 sequences. As mentioned above, the indicated lentiviruses from different groups were harvested and transduced into PANC-1 cells. The human miR-1294 inhibitor plasmid was purchased from Genomeditech (Shanghai, China). Then, the control-shRNA, sh-circEYA3, sh-circEYA3 and the miR-1294 inhibitor were stably transduced into PANC-1 cells and selected with puromycin for 2 weeks. For the tumor growth study, male BALB/c athymic nude mice (4 weeks old) were obtained from Shanghai SLAC Laboratory (Shanghai, China) and inoculated with cells to generate xenograft tumors (*n* = 5 mice/group). Stably transfected PANC-1 cells (3 × 10^6^) in 100 μL of PBS were inoculated subcutaneously into the right flanks of the mice. Tumor growth was monitored weekly. Tumors were measured weekly with callipers, and tumor volumes were calculated with the following equation: volume = 0.5 × length×width^2^. Thirty-eight days after cell inoculation, the body weight were measured, and the tumors were excised, weighed and fixed with 4% paraformaldehyde for IHC staining.

### Statistical analysis

GraphPad Prism 8.0 and SPSS 23.0 were applied for statistical analysis. Student’s t-test was utilized to analyse differences between two groups, and one-way ANOVA was used for multi-group comparisons. The significance of associations between the levels of circEYA3 and miR-1294 and clinicopathologic parameters was determined by the χ2 test and Fisher’s exact test. Correlations were evaluated by Pearson correlation analysis. Survival was analysed by Kaplan-Meier method, and significance was evaluated with the log-rank test. A *P*-value of < 0.05 was considered statistically significant for all tests. The results are shown as the mean ± SD of at least three independent experiments.

## Results

### Discovery of oncogenic circRNAs and characterization of circEYA3 in PDAC

**Fig. 1:**
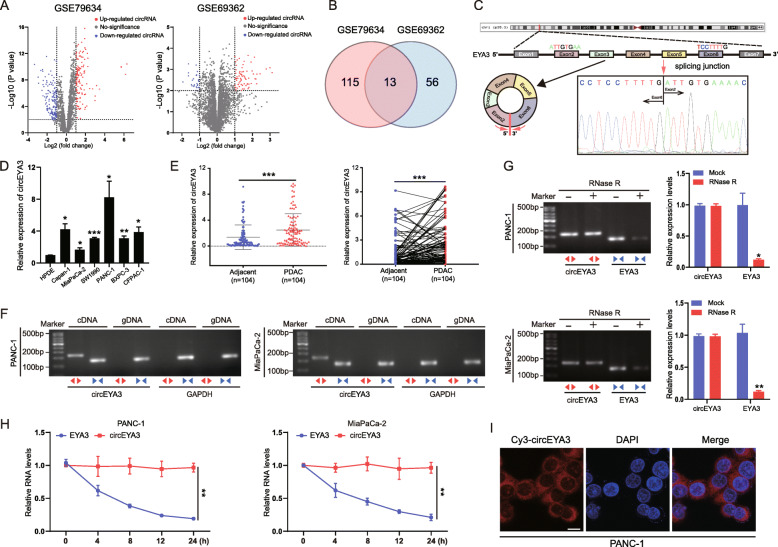
Discovery of oncogenic circRNAs and characterization of circEYA3 in PDAC **A**. Volcano plots showing the upregulated (red) and downregulated (blue) circRNAs in PDAC from GSE79634 and GSE69362 (fold change > 2 or < 0.5, *P* < 0.01). **B** Venn diagram exhibiting the overlap of the upregulated circRNAs in GSE79634 and GSE69362. **C** Schematic illustration presenting the formation of circEYA3 from exons 2–6 of the EYA3 gene. Sanger sequencing verified the existence of circEYA3. The red arrow indicates the back-splicing junction sites in exons 2 and 6 of the EYA3 gene. **D** The relative expression level of circEYA3 in PDAC cells and HPDE cells was determined by qRT-PCR. **E** qRT-PCR was used to evaluate the expression of circEYA3 in paired PDAC and matched adjacent normal tissues (*n* = 104). **F** Divergent (red) and convergent (blue) primers were used to amplify the back-spliced and linear products to verify the existence of circEYA3 using PANC-1 and MiaPaCa-2 cells. **G** qRT-PCR and subsequent nucleic acid electrophoresis were used to analyse the expression of circEYA3 and EYA3 mRNA after treatment with RNase R. **H** Alterations in the circEYA3 and EYA3 mRNA levels were analysed by qRT-PCR under treatment with the transcription inhibitor ActD. **I** FISH was conducted to detect the distribution of circEYA3 in PDAC cells. Red fluorescence (Cy3-labelled probe) indicates circEYA3. Nuclei were stained with DAPI (blue). Scale bar, 10 μm. All data are shown as the mean ± SD values. *P < 0.05, **P < 0.01, ***P < 0.001

To search for potential circRNAs involved in PDAC progression, we systematically analysed the differentially expressed circRNAs in PDAC tissues compared with adjacent noncancerous tissues in two open-access published GEO datasets (GSE79634 and GSE69362) [23, 24]. The volcano plot shows that several circRNAs in GSE79634 and GSE69362 were upregulated or downregulated according to the following criteria: fold change > 2 or < 0.5 and *P* < 0.01 (Fig. 1A). Among the differentially expressed circRNAs, upregulated circRNAs were more common than downregulated circRNAs in GSE69362. In addition, many previous studies have highlighted that some circRNAs are upregulated in PDAC cells and tissues and perform oncogenic functions in PDAC [13, 25, 26]. Thus, we focused on the upregulated circRNAs in PDAC. The intersection of the upregulated circRNAs in GSE79634 and GSE69362 visualized in a Venn diagram indicated that 13 circRNAs (hsa_circ_0064288, hsa_circ_0007367, hsa_circ_0007767, hsa_circ_0060733, hsa_circ_0049783, hsa_circ_0092310, hsa_circ_0029634, hsa_circ_0007895, hsa_circ_0006117, hsa_circ_0074903, hsa_circ_0008253, hsa_circ_0047585, and hsa_circ_0066147) were upregulated in both datasets (Fig. 1B). Subsequently, we selected the 3 most upregulated candidate circRNAs (hsa_circ_0029634, hsa_circ_0006117, and hsa_circ_0007895) for further study. Next, the expression levels of these three circRNAs were determined by qRT-PCR in 20 randomly selected pairs of PDAC tissues and matched adjacent normal tissues. The results of qRT-PCR and GEO dataset analysis showed that the level of hsa_circ_0007895 was prominently increased in PDAC tissues compared with adjacent normal tissues, and hsa_circ_0007895 was thus selected for further study (Fig. S1A and S1B). Hsa_circ_0007895 is produced from exons 2–6 of the human EYA3 gene on chromosome 1p35.3 and contains 492 nucleotides (Fig. 1C). Thus, we termed hsa_circ_0007895 “circEYA3” in this article. Next, we observed that the level of circEYA3 was significantly higher in several PDAC cell lines than in the HPDE cell line (Fig. 1D). Among these cell lines, PANC-1 exhibited the highest level and MiaPaCa-2 exhibited the lowest level of circEYA3 (Fig. 1D). Next, to explore the relationship between the level of circEYA3 and the clinical characteristics of PDAC patients, qRT-PCR was used to determine the expression of circEYA3 in a cohort comprising 104 pairs of PDAC tumor and matched adjacent normal tissues. As shown in Fig. 1E, circEYA3 was obviously upregulated in the PDAC tissues compared with the matched adjacent normal tissues. Additionally, high expression of circEYA3 was correlated with advanced lymph node invasion and tumour-node-metastasis (TNM) stage (Additional file 1: TableS5). These results indicated that higher circEYA3 expression may be related to the pathogenesis of PDAC. However, no significant correlations were found between the circEYA3 expression level and other features, such as age, sex, tumor grade, tumor location, tumor stage, tumor size, and CA19–9 level.

To prove that circEYA3 is a novel circular rather than linear structure, divergent and convergent primers specific for circEYA3 and the corresponding linear transcript were designed. Then, using cDNA and gDNA from PANC-1 and MiaPaCa-2 cells as templates, we found that circEYA3 was amplified using the divergent primers (an expected 168-bp fragment) only from cDNA; no amplification product was detected from gDNA (Fig. 1F). Next, we conducted Sanger sequencing to verify back-splicing sites in the qRT-PCR product of circEYA3 (Fig. 1C). To verify the stability of circEYA3, the extracted RNA was treated with RNase R, and the circular form (circEYA3) was resistant to digestion by RNase R in both PANC-1 and MiaPaCa-2 cells; however, the level of the linear form (EYA3 mRNA) was greatly decreased (Fig. 1G). In addition, ActD (a transcription inhibitor) was utilized to confirm the stability of circEYA3, and circEYA3 was found to be more stable than EYA3 mRNA (Fig. 1H). Furthermore, RNA FISH showed that circEYA3 was localized primarily in the cytoplasm (Fig. 1I). Collectively, these data identified circEYA3 as an abundant and stable transcript in PDAC.

### CircEYA3 facilitates the proliferation, migration, and invasion and inhibits the apoptosis of PDAC cells in vitro

**Fig. 2:**
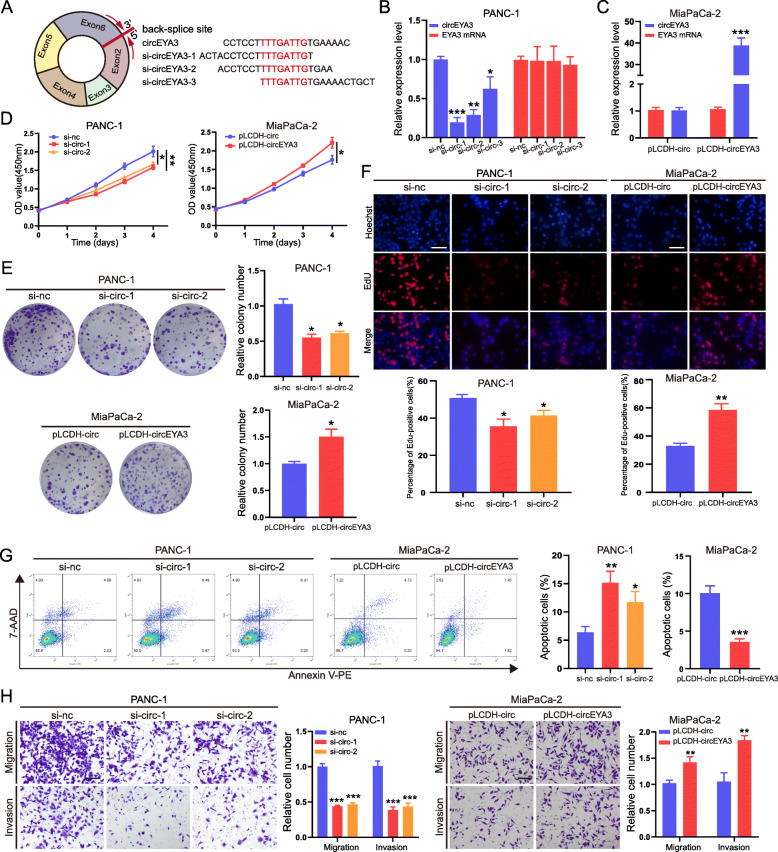
CircEYA3 facilitates the proliferation, migration, and invasion and inhibits the apoptosis of PDAC cells in vitro. **A** Three siRNAs targeting the circEYA3 junction site were constructed. **B** The expression of circEYA3 and EYA3 mRNA in PANC-1 cells was analysed by qRT-PCR after transfection with three siRNAs or the control siRNA (si-NC). **C** The levels of circEYA3 and EYA3 mRNA in MiaPaCa-2 cells were analysed by qRT-PCR after stable transfection with the circEYA3 overexpression vector (pLCDH-circEYA3) or the control vector (pLCDH-circ). **D**, **E** and **F**. Cell proliferation was assessed by CCK-8 (**D**), colony formation (**E**), and EdU incorporation assays (**F**). Scale bars, 100 μm. Downregulation of circEYA3 significantly inhibited the proliferation of PANC-1 cells, while ectopic upregulation of circEYA3 promoted the proliferation of MiaPaCa-2 cells. **G.** Apoptosis of PDAC cells was evaluated by Annexin V-PE/7-AAD staining when circEYA3 was downregulated and upregulated. **H**. Transwell assays were used to evaluate the migratory and invasive capabilities of PDAC cells when circEYA3 was downregulated or upregulated. Scale bars, 100 μm. *P < 0.05, **P < 0.01, ***P < 0.001

Next, to evaluate the biological roles of circEYA3 in the behaviours of PDAC cells, three siRNAs were designed and constructed to target the back-spliced region of circEYA3 to specifically downregulate its expression (Fig. 2A). After PANC-1 cells were transfected with the three siRNAs, the qRT-PCR results indicated that all three siRNAs effectively silenced circEYA3 without altering the expression of its host linear EYA3 mRNA and that siRNA #1 and #2 had the highest knockdown efficiency (Fig. 2B). In addition, circEYA3 was successfully stably overexpressed by transfection of recombinant circEYA3 plasmids in MiaPaCa-2 cells, and no significant changes were observed in the level of EYA3 mRNA (Fig. 2C). The CCK-8 assay results showed that circEYA3 knockdown efficiently decreased the proliferation and viability of PANC-1 cells at 96 h post transfection, while upregulation of the target circEYA3 obviously enhanced the proliferation ability of MiaPaCa-2 cells (Fig. 2D). The colony formation assay further indicated that the number of colonies formed by PANC-1 cells was significantly reduced by knockdown and increased by overexpression of circEYA3 (Fig. 2E). In addition, the EdU incorporation assay as a measure of cell proliferation showed that the percentages of EdU-positive cells were greatly decreased by knockdown but increased by overexpression of circEYA3 (Fig. 2F). To further explore the biological function of circEYA3, apoptosis of PANC-1 and MiaPaCa-2 cells was evaluated by Annexin V-PE/7-AAD staining. The apoptosis rate was increased by downregulation of circEYA3 in PANC-1 cells but decreased by overexpression of circEYA3 in MiaPaCa-2 cells (Fig. 2G). Moreover, the Transwell assay showed that downregulation of circEYA3 obviously decreased the migratory and invasive abilities of PANC-1 cells, while upregulation of circEYA3 led to the opposite effects in MiaPaCa-2 cells (Fig. 2H). In the 3D spheroid invasion assays, we also observed that downregulation of circEYA3 inhibited tumour spheroid invasion in PANC-1 cells, while overexpression of circEYA3 increased spheroid invasion in MiaPaCa-2 cells (Fig. S2A and S2B). Taken together, the results of these experiments indicated that circEYA3 performs an oncogenic function in PDAC cells.

### CircEYA3 acts as an efficient miR-1294 sponge in PDAC

**Fig. 3:**
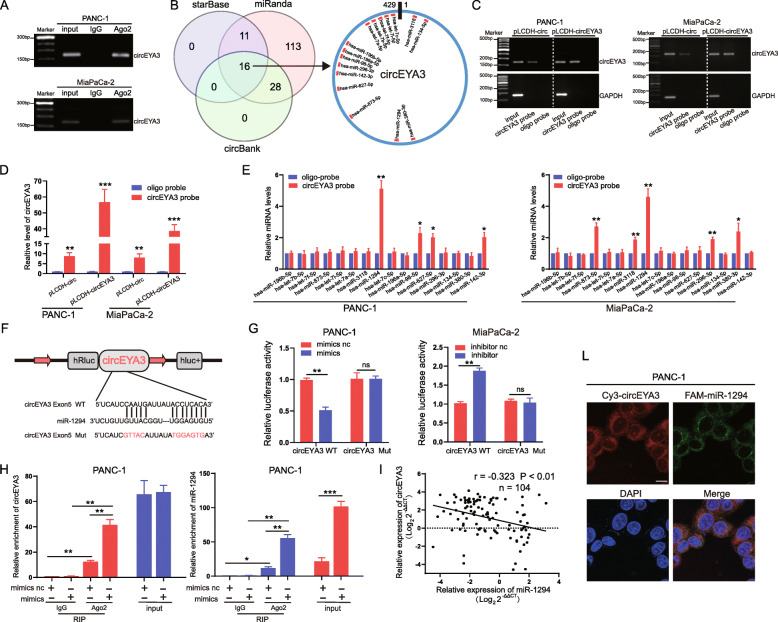
CircEYA3 acts as an efficient miR-1294 sponge in PDAC. **A**. The level of circEYA3 was analysed by a RIP assay in the anti-Ago2 antibody immunoprecipitate from PDAC cells. **B** The potential target miRNAs of circEYA3 were predicted in the circBank, starBase and miRanda databases. **C** and **D**. CircEYA3 in PANC-1 and MiaPaCa-2 cell lysates was pulled down and enriched with a specific circEYA3 probe. qRT-PCR and gel electrophoresis were used to determine the specificity and efficiency of the circEYA3 probe. **E**. qRT-PCR was utilized to determine the relative expression levels of 16 potential target miRNAs in precipitates from PANC-1 and MiaPaCa-2 cell lysates pulled down by the circEYA3 probe or oligo probe. **F**. Schematic illustration of the circEYA3-WT and circEYA3-Mut luciferase vectors. **G** Relative luciferase activities in PANC-1 and MiaPaCa-2 cells co-transfected with circEYA3-WT or circEYA3-Mut and the miR-1294 mimic, inhibitor or corresponding negative control. **H** A RIP assay was carried out with anti-Ago2 antibodies or IgG in PANC-1 cells after transfection with the miR-1294 mimic or mimic NC, and qRT-PCR was then performed to detect the enrichment of circEYA3 and miR-1294. (I) The correlation between the circEYA3 and miR-1294 expression levels in 104 paired PDAC patients was analysed by RT-qPCR and Pearson correlation analysis. **L** FISH assay was used to observe the cellular location of circEYA3 (red) and miR-1294 (green). Scale bar, 10 μm. *P < 0.05, **P < 0.01, ***P < 0.001; ns indicates no significance

**Fig. 4:**
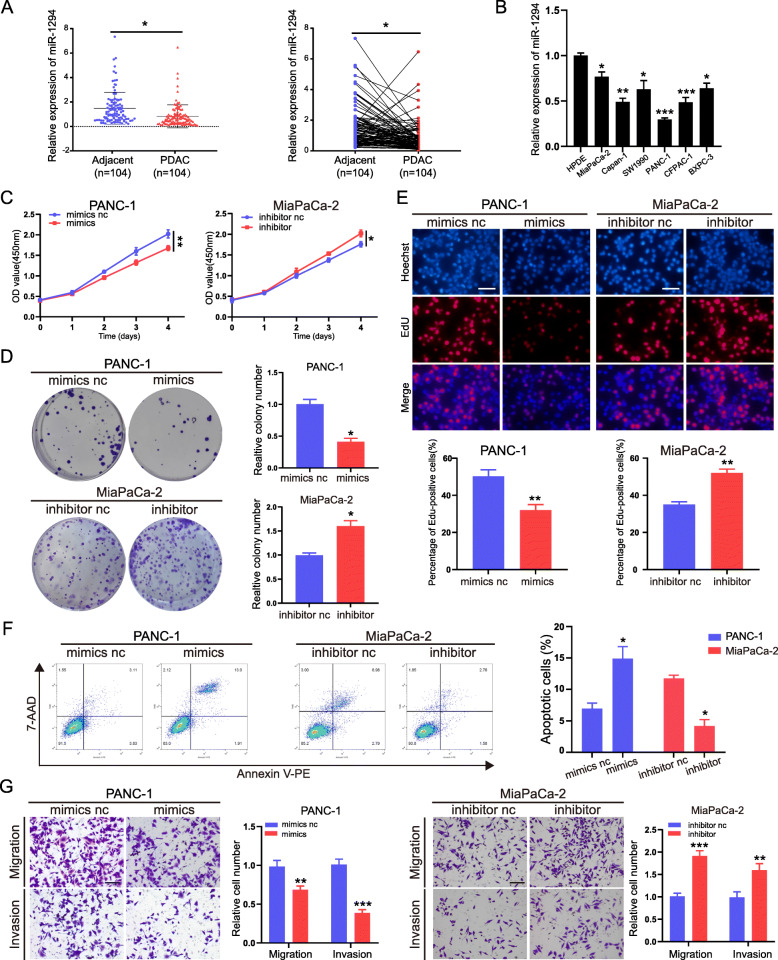
miR-1294 inhibits the proliferation, migration, and invasion and promotes the apoptosis of PDAC cells in vitro*.* **A** and **B**. qRT-PCR was performed to evaluate the expression of miR-1294 in paired PDAC and matched adjacent normal tissues (*n* = 104) (A) and in PDAC cell lines and an HPDE cell line (B). **C**, **D** and **E**. Cell proliferation was assessed by CCK-8 (C), colony formation (D), and EdU incorporation assays (E). Scale bars, 100 μm. Upregulation of miR-1294 significantly inhibited the proliferation of PANC-1 cells, while ectopic downregulation of circEYA3 promoted the proliferation of MiaPaCa-2 cells. **F**. Apoptosis of PDAC cells was evaluated by Annexin V-PE/7-AAD staining when miR-1294 was upregulated or downregulated. **H**. A Transwell assay was performed to evaluate the migratory and invasive capabilities of PDAC cells when miR-1294 was upregulated or downregulated. Scale bars, 100 μm. *P < 0.05, **P < 0.01, ***P < 0.001

Existing studies have demonstrated that many circRNAs exert their biological effects by sponging miRNAs and subsequently regulating miRNA expression in PDAC [13, 14, 25]. Given that circEYA3 exhibited high stability and was localized mainly in the cytoplasm, as described above, we hypothesized that circEYA3 facilitated malignant biological behaviours of PDAC cells by sponging miRNAs. Therefore, to validate this hypothesis, RIP assays using an anti-Ago2 antibody and IgG were performed in PANC-1 cells and MiaPaCa-2 cells. We found that endogenous circEYA3 was pulled down by anti-Ago2, indicating that circEYA3 bound to miRNAs via the Ago2 protein (Fig. 3A). Subsequently, three databases (starBase, miRanda, and circBank) were used for bioinformatic analysis to predict the potential target miRNAs of circEYA3. Then, 16 candidate miRNAs were identified from the intersection of the miRNAs identified in these three databases, as shown in Fig. 3B. RNA pull-down experiments were subsequently performed using a biotin-labelled probe specific for circEYA3 and a random oligo negative control probe in PANC-1 and MiaPaCa-2 cells transfected with the pLCDH-circEYA3 overexpression plasmid and the corresponding pLCDH-circ control plasmid. A greater amount of circEYA3 was pulled down in both cell lines after transfection with the pLCDH-circEYA3 overexpression plasmid (Fig. 3C and D). The expression levels of the 16 candidate miRNAs in the immunoprecipitates were determined using a biotin-labelled probe specific for circEYA3. The qRT-PCR results indicated that miR-1294 was the only miRNA that was statistically abundant in both PANC-1 and MiaPaCa-2 cells, suggesting that miR-1294 specifically interacted with circEYA3 (Fig. 3E). Next, luciferase reporter plasmids containing WT circEYA3 and circEYA3 with a mutated miR-1294 binding site were constructed (Fig. 3F). The luciferase activity of WT circEYA3 was markedly suppressed in PANC-1 cells transfected with the miR-1294 mimic but elevated in MiaPaCa-2 cells transfected with the miR-1294 inhibitor. Conversely, the luciferase activity of the circEYA3 mutant was not significantly changed among the miR-1294 mimic, miR-1294 inhibitor and corresponding negative control groups, indicating the possibility of a direct interaction between miR-1294 and circEYA3 (Fig. 3G). To further validate this interaction, a RIP assay using an anti-AGO2 antibody and negative control IgG was performed in PANC-1 cells transiently overexpressing miR-1294 to pull down circEYA3 and miR-1294, and qRT-PCR was then performed to analyse the levels of circEYA3 and miR-1294 levels in the precipitates. Both circEYA3 and miR-1294 were pulled down in significantly higher quantities by the anti-Ago2 antibody compared with IgG (Fig. 3H). Furthermore, circEYA3 and miR-1294 were dramatically more abundant in cells transfected with the miR-1294 mimic compared with cells transfected with the mimic negative control (mimic NC) (Fig. 3H). Next, the expression level of miR-1294 was determined by qRT-PCR in 104 pairs of PDAC tumor and paracarcinoma tissues, and miR-1294 expression was found to be obviously downregulated in PDAC tissues (Fig. 4A). Pearson correlation analysis indicated that miR-1294 expression was negatively associated with circEYA3 expression, as determined by qRT-PCR, in these 104 pairs of fresh frozen PDAC tissues (Fig. 3I). We also found that downregulation of circEYA3 increased miR-1294 expression, while upregulation of circEYA3 decreased miR-1294 expression (Fig. S3A). Furthermore, the FISH results confirmed the co-localization of circEYA3 and miR-1294 mainly in the cytoplasm of PANC-1 cells (Fig. 3L). Taken together, these findings indicated that circEYA3 can directly bind with miR-1294 in PDAC cells.

### MiR-1294 inhibits the proliferation, migration, and invasion and promotes the apoptosis of PDAC cells in vitro

Reports have confirmed that miR-1294 acts as a tumor suppressor in different cancers [27–29]. A study by Yi et al. also indicated that miR-1294 expression was lower in PDAC tissue samples than in the paired adjacent normal tissues, as determined by qRT-PCR [30], consistent with our analysis of a 104-case cohort of fresh frozen PDAC tissues (Fig. 4A). However, no obvious relationships were observed between miR-1294 expression and PDAC clinicopathological features based on our qRT-PCR results (Additional file 1: Table S5). To determine the specific biological roles of miR-1294 in PDAC, we further explored its functions in PDAC cells. qRT-PCR analysis showed that miR-1294 expression was apparently lower in PDAC cells than in HPDE cells. Among the tested cell lines, PANC-1 exhibited the lowest level and MiaPaCa-2 cells exhibited the highest level of miR-1294 (Fig. 4B). Thus, we selected PANC-1 and MiaPaCa-2 cells for further research. PANC-1 and MiaPaCa-2 cells were transfected with the miR-1294 mimic or mimic NC or with the miR-1294 inhibitor or inhibitor NC, respectively. Then, the transfection efficiency was confirmed by qRT-PCR (Fig. S3B and S3C). The CCK-8 and colony formation assays showed that upregulated expression of miR-1294 inhibited PANC-1 cell proliferation, whereas downregulated expression of miR-1294 led to the opposite effects (Fig. 4C and D). Consistent with these findings, the EdU incorporation assay showed that the percentage of EdU-positive cells was significantly decreased by upregulation of miR-1294 but increased by downregulation of miR-1294 (Fig. 4E). Moreover, the apoptosis assay showed that upregulation of miR-1294 promoted the apoptosis of PANC-1 cells, whereas downregulation of miR-1294 exerted the opposite effect on MiaPaCa-2 cells (Fig. 4F). The Transwell assay showed that miR-1294 mimic transfection notably decreased the migratory and invasive abilities of PANC-1 cells, while miR-1294 inhibitor transfection resulted in the opposite trends in MiaPaCa-2 cells (Fig. 4G). These results demonstrated that miR-1294 may function as a tumor suppressor in PDAC in vitro.

### C-Myc is a direct target of miR-1294 in PDAC cells

**Fig. 5:**
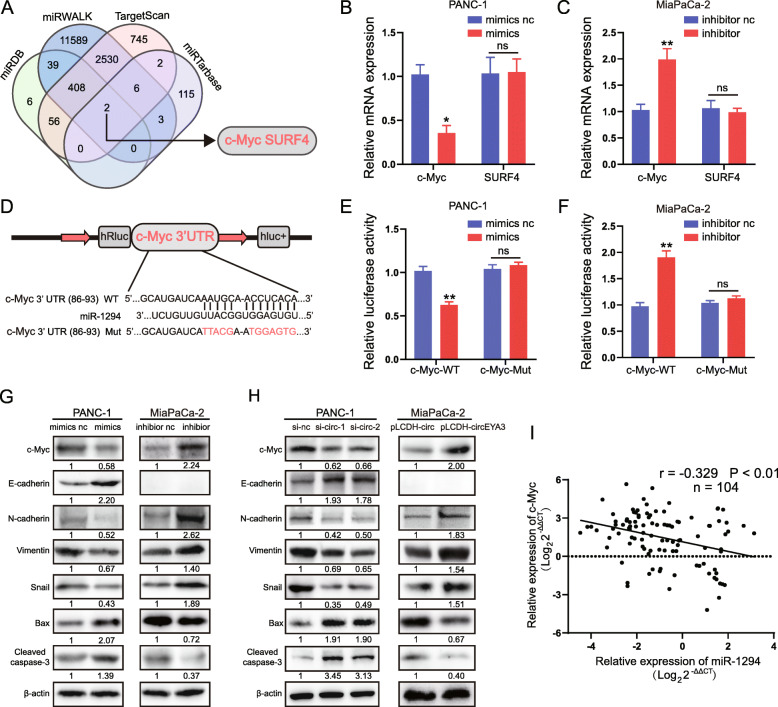
c-Myc is a direct target of miR-1294 in PDAC cells. **A**. The miRDB, miRWalk, TargetScan, and miRTarBase databases were utilized to predict the target genes of miR-1294. **B** and **C**. PANC-1 and MiaPaCa-2 cells were transfected with the miR-1294 mimic, miR-1294 inhibitor or the corresponding negative control, and the expression of c-Myc and SURF4 was evaluated by qRT-PCR. **D**. Schematic showing the c-Myc-WT and c-Myc-Mut luciferase reporter plasmids. **E** and **F**. Relative luciferase activity in PANC-1 and MiaPaCa-2 cells after co-transfection with c-Myc-WT or c-Myc-Mut and the miR-1294 mimic, inhibitor or corresponding negative control. **G** and **H**. Western blot analysis was performed to assess the protein levels of c-Myc, E-cadherin, N-cadherin, Vimentin, Snail, Bax, and cleaved caspase-3 after knocking down or overexpressing miR-1294 or circEYA3 in PANC-1 and MiaPaCa-2 cells, as indicated. β-actin was used as the loading control. **I**. The correlation between the miR-1294 and c-Myc expression levels in 104 paired PDAC patients was analysed by qRT-PCR and Pearson correlation analysis. *P < 0.05, **P < 0.01, ***P < 0.001; ns indicates no significance

To further investigate the downstream genes of miR-1294 in PDAC cells, the miRDB, miRWalk, TargetScan and miRTarBase databases were applied to identify the potential downstream target genes of miR-1294. Among the identified potential target genes, two (c-Myc and SURF4) overlapped in the data obtained from these four databases (Fig. 5A). Then, we transfected the miR-1294 mimic and inhibitor into PANC-1 cells and MiaPaCa-2 cells, respectively, to detect whether the levels of the predicted miR-1294 target genes were altered. The qRT-PCR results showed that upregulation of miR-1294 decreased the expression of c-Myc in the miR-1294 mimic group but had no effect on the expression of SURF4 (Fig. 5B). Similarly, downregulation of miR-1294 increased the expression of c-Myc (Fig. 5C). In fact, previous studies have proven that miR-1294 can directly target the 3’UTR of c-Myc and function as a tumor suppressor in oesophageal squamous cell carcinoma (ESCC) and oral squamous cell carcinoma (OSCC) [31, 32]. Hence, to validate whether miR-1294 directly targets c-Myc in PDAC cells, dual-luciferase reporter assays were performed, and cells co-transfected with c-Myc-WT and the miR-1294 mimics or inhibitor exhibited dramatically reduced or increased luciferase activity, respectively, while the luciferase activity was not significantly altered in cells co-transfected with c-Myc-Mut and the miR-1294 mimic or inhibitor (Fig. 5C and D). In line with the roles of miR-1294 in inhibiting PDAC progression, western blot analysis showed that overexpression of miR-1294 markedly reduced the expression of c-Myc, N-cadherin, Vimentin, and Snail and elevated the expression of E-cadherin, Bax, and cleaved caspase-3; downregulation of miR-1294 exerted the opposite effects (Fig. 5G). Moreover, in line with the roles of circEYA3 in promoting PDAC progression, downregulation of circEYA3 reduced the expression of c-Myc, N-cadherin, Vimentin, and Snail and elevated the expression of E-cadherin, Bax, and cleaved caspase-3, whereas overexpression of circEYA3 exerted opposite effects (Fig. 5H). Moreover, c-Myc expression was negatively correlated with miR-1294 expression in our previous cohort of PDAC tissues (Fig. 5I). The data presented herein indicated that miR-1294 can directly bind to the 3′ UTR of c-Myc to repress its expression in PDAC cells.

### CircEYA3 performs its oncogenic functions by increasing ATP production through the miR-1294/c-Myc axis

**Fig. 6:**
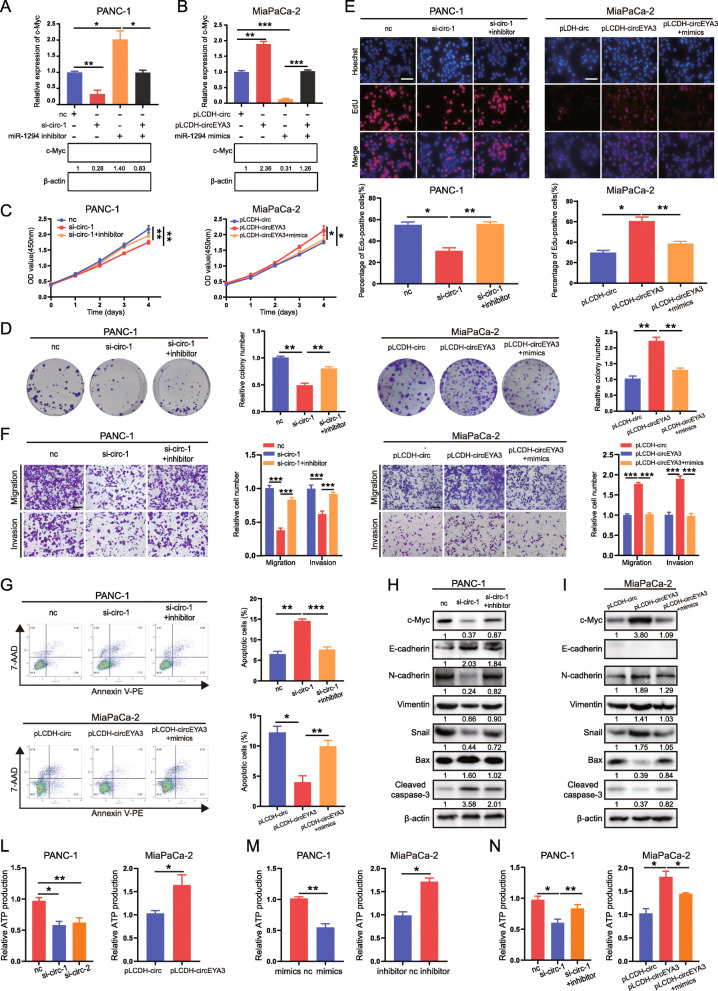
CircEYA3 performs its oncogenic functions by increasing ATP production through the miR-1294/c-Myc axis. **A** and **B**. PANC-1 cells were transfected with NC, si-circ-1 and the miR-1294 inhibitor, and MiaPaCa-2 cells were transfected with NC, pLCDH-circEYA3 and the miR-1294 mimics, as indicated. qRT-PCR and western blot analysis were performed to assess the expression of c-Myc. **C**, **D** and **E**. The cell proliferation ability was evaluated by CCK-8 (**C**), colony formation (**D**), and EdU incorporation assays (**E**). Scale bars, 100 μm. The miR-1294 inhibitor significantly reversed the reduction in cell proliferation caused by circEYA3 knockdown in PANC-1 cells, while the miR-1294 mimic further enhanced the proliferation of MiaPaCa-2 cells. **F**. The effects of si-circ-1 and miR-1294 or pLCDH-circEYA3 and the miR-1294 mimic on the migratory and invasive capabilities of PDAC cells were evaluated by Transwell assays. Scale bars, 100 μm. **G**. The effects of si-circ-1 and miR-1294 or pLCDH-circEYA3 and the miR-1294 mimic on apoptosis were evaluated by Annexin V-PE/7-AAD staining. **H** and **I**. Western blot analysis was performed to determine the protein levels of c-Myc, E-cadherin, N-cadherin, Vimentin, Snail, Bax, and cleaved caspase-3 in order to evaluate the effects of si-circ-1 and miR-1294 or pLCDH-circEYA3 and the miR-1294 mimic on PANC-1 (**H**) or MiaPaCa-2 (**I**) cells, respectively. **L**. CircEYA3 knockdown decreased and circEYA3 overexpression increased ATP production. **M**. The miR-1294 mimic decreased but the miR-1294 inhibitor induced ATP production. **N**. The effects of si-circ-1 and the miR-1294 inhibitor or pLCDH-circEYA3 and the miR-1294 mimic on ATP production in PANC-1 and MiaPaCa-2 cells. *P < 0.05, **P < 0.01, ***P < 0.001

As previously mentioned, altering the expression of circEYA3 significantly affected the expression of c-Myc. To verify whether circEYA3 promoted the progression of PDAC cells through the miR-1294/c-Myc axis, we simultaneously co-transfected si-circEYA3–1 and the miR-1294 inhibitor or inhibitor NC into PANC-1 cells and co-transfected the pLCDH-circEYA3 overexpression plasmid and the miR-1294 mimic or mimic NC into MiaPaCa-2 cells for rescue experiments. Western blot analysis and qRT-PCR showed that the downregulation of circEYA3 decreased c-Myc protein expression and that the miR-1294 inhibitor rescued protein expression, while upregulation of circEYA3 and transfection with the miR-1294 mimic caused the opposite effects (Fig. 6A and B). Additionally, circEYA3 expression was positively correlated with c-Myc expression based on our previous qRT-PCR results (Fig. S4A). As shown in Fig. 6D-G, a series of in vitro functional experiments revealed that the biological effects of circEYA3 were reversed by the miR-1294 mimic or inhibitor. The results of the CCK-8 assays, EdU proliferation, colony formation, apoptosis, and Transwell assays showed that the miR-1294 inhibitor reversed the alterations in the biological behaviours of PDAC cells induced by knockdown of circEYA3. Conversely, the miR-1294 mimic offset the alterations in the biological behaviours of PDAC cells induced by overexpression of circEYA3 (Fig. 6D-G). Subsequent western blot analysis also confirmed that the miR-1294 inhibitor and mimic reversed the alterations in EMT- and apoptosis related protein levels caused by alterations in circEYA3 (Fig. 6H and I).

Our previous study confirmed that in pancreatic cancer, c-Myc can increase energy metabolism/ATP production, which is essential for many cellular processes [22]. Therefore, we investigated whether circEYA3 performs its biological functions by affecting the cellular production of ATP. Specifically, downregulation of circEYA3 significantly decreased the production of ATP in PANC-1 cells, while upregulation of circEYA3 exerted the opposite effect in MiaPaCa-2 cells (Fig. 6L). As expected, downregulation of miR-1294 also markedly increased but upregulation of miR-1294 decreased the production of ATP (Fig. 6M). Furthermore, the miR-1294 inhibitor and mimic considerably reversed the alterations in ATP production caused by downregulation and upregulation of circEYA3, respectively (Fig. 6N). Specifically, these findings collectively demonstrated that circEYA3 may increase the production of ATP to perform its oncogenic biological functions via the miR-1294/c-Myc axis.

### Knockdown of circEYA3 suppresses tumor growth from PDAC cells in vivo

**Fig. 7:**
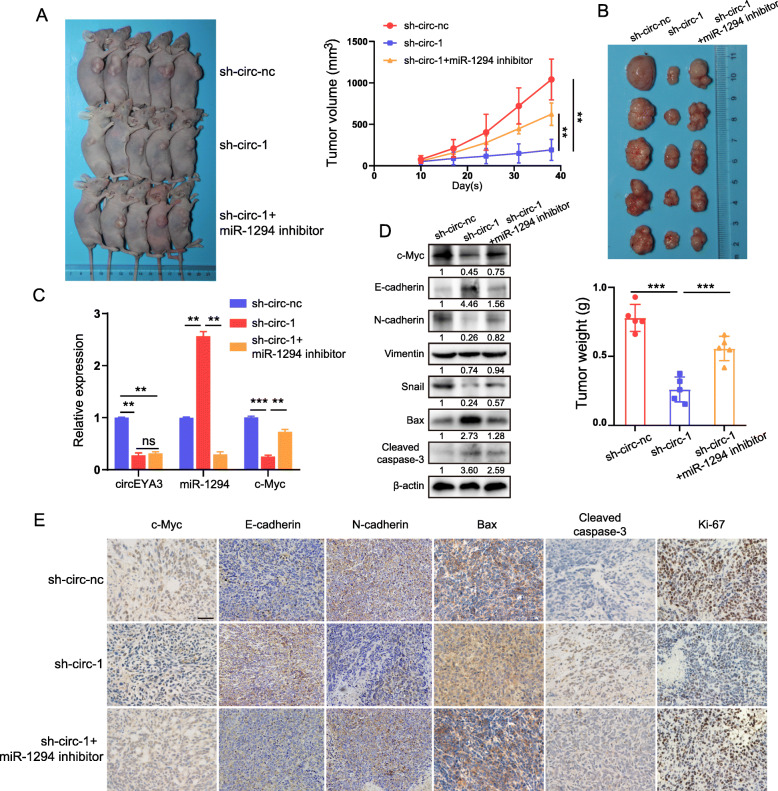
Knockdown of circEYA3 suppresses tumor growth in vivo. **A.** Stably transfected PANC-1 cells from different groups were inoculated into BALB/c nude mice to establish subcutaneous xenograft tumors (*n* = 5 mice/group). Representative images of tumor-bearing mice. In addition, tumor volumes were monitored weekly. **B**. The excised tumors were photographed and weighed. **C**. The relative expression levels of circEYA3, miR-1294 and c-Myc in subcutaneous tumor tissues were determined by qRT-PCR. **D**. The c-Myc, E-cadherin, N-cadherin, Vimentin, Snail, Bax, cleaved caspase-3 protein levels in tumours from different groups were determined by western blot analysis. **E**. IHC staining showed the relative levels of c-Myc, E-cadherin, N-cadherin, Bax, cleaved caspase-3, and Ki-67 in tumours from different groups. Scale bar, 50 μm. *P < 0.05, **P < 0.01, ***P < 0.001; ns indicates no significance

To further explore the potential role of circEYA3 and miR-1294 in vivo, cells were stably transfected with either sh-circ-nc or sh-circEYA3–1 or co-transfected with sh-circEYA3–1 and the miR-1294 inhibitor to establish the xenograft model. The in vivo data confirmed that the tumor volumes and weights were significantly decreased by knockdown of circEYA3 (Fig. 7A and B). Unexpectedly, the miR-1294 inhibitor significantly counteracted the reductions in the tumor volumes and weights induced by knockdown of circEYA3 (Fig. 7A and B). However, there was no significant difference in body weight between the groups (Fig. S4B). Total RNA and protein were extracted from the tumors in each group. Subsequent qRT-PCR showed that circEYA3 knockdown induced major decreases in the mRNA levels of c-Myc and miR-1294 (Fig. 7C). Notably, we observed that the miR-1294 inhibitor largely counteracted this effect (Fig. 7C). Moreover, after knockdown of circEYA3 expression, the protein levels of c-Myc, N-cadherin, Vimentin, and Snail obviously decreased, while those of E-cadherin, Bax and cleaved caspase-3 markedly increased, and the miR-1294 inhibitor partially reversed these alterations (Fig. 7D). Moreover, IHC staining with anti-c-Myc, anti-E-cadherin, anti-N-cadherin, anti-Bax, anti-cleaved caspase-3, and anti-Ki-67 antibodies was performed on xenograft tumour tissues. The IHC results indicated that circEYA3 knockdown induced decreases in the expression of c-Myc, N-cadherin and Ki-67 but increases in the levels of E-cadherin, Bax and cleaved caspase-3 (Fig. 7E). More importantly, the miR-1294 inhibitor again counteracted these alterations (Fig. 7E). These findings demonstrated that knockdown of circEYA3 inhibits the growth of PDAC tumours in vivo, consistent with the in vitro results.

### Overexpression of circEYA3 is associated with poor prognosis in PDAC patients

**Fig. 8:**
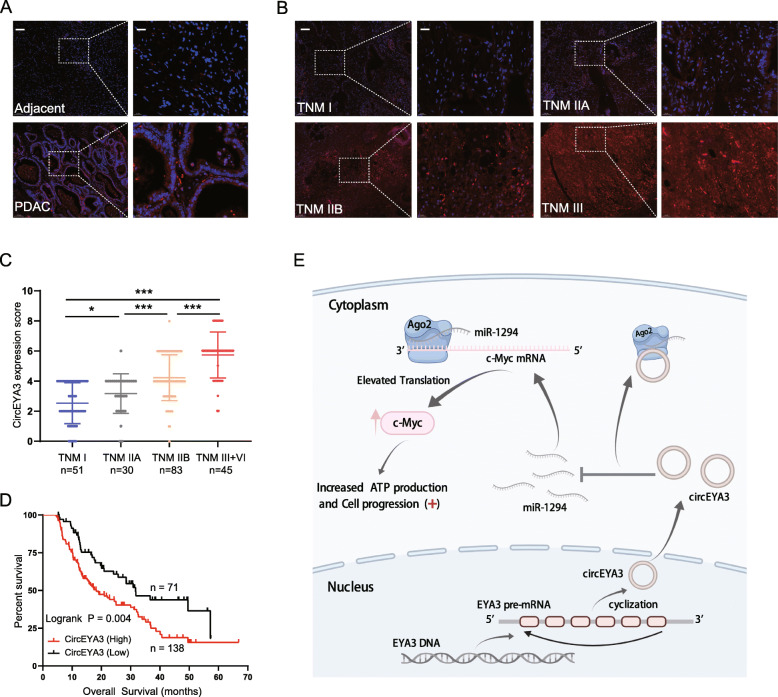
Overexpression of circEYA3 is associated with poor prognosis in PDAC patients. **A** and **B**. Representative FISH images of circEYA3 expression in PDAC tissues and adjacent normal tissues, as well as in tissues from tumors with different TNM stages. Nuclei were stained with DAPI. Left panel, scale bar, 100 μm; right panel, scale bar 20 μm. **C**. CircEYA3 FISH scores in tissues from tumors with different TNM stages. **D**. Kaplan-Meier survival analysis of circEYA3 expression in PDAC patients based on our data (*n* = 209, *P* = 0.004, log-rank test). **E**. Schematic illustration of the circEYA3/miR-1294/c-Myc axis in PDAC cells. * *P* < 0.05, **P < 0.01, ***P < 0.001

To further investigate the clinical significance of circEYA3 in PDAC, FISH was performed on TMAs containing 209 PDAC patients and adjacent normal tissues. The FISH results indicated that circEYA3 expression was markedly upregulated in PDAC tissues compared with adjacent normal tissues (Fig. 8A). Analysis of the clinical characteristics of patients with PDAC revealed that circEYA3 expression was increased with advanced TNM stage (Fig. 8B and C; Table 1). Interestingly, the circEYA3 expression level was significantly correlated with the CA19–9 level; the higher the expression of circEYA3 was, the higher the level of CA19–9 (Table 1). However, no correlations were found between circEYA3 expression and age, sex, tumor location or histological grade (Table 1). Notably, Kaplan-Meier survival analysis revealed that PDAC patients with high circEYA3 expression had poorer clinical outcomes than those with low circEYA3 expression (Fig. 8D). Collectively, these results suggested that overexpression of circEYA3 may predict poor prognosis in PDAC patients.

**Table 1:** Correlation between circEYA3 and clinicopathological parameters in PDAC form TMA (*n* = 209)

Characteristic	Total (209)	circEYA3 expression		*P* value
		Low (71)	High (138)	
Age				
≤ 60	94	30	64	
> 60	115	41	74	0.570
Sex				
Male	105	30	75	
Female	104	41	63	0.098
Tumor location				
Head	64	21	43	
Body/tail	145	50	95	0.814
Grade				
High/Moderate	142	52	90	
Low	67	19	48	0.239
TNM stage				
I	51	32	19	
IIA	30	14	16	
IIB	83	22	61	
III + VI	45	3	42	0.000***
CA19–9 level				
≤ 37 U/mL	44	25	19	
> 37 U/mL	165	46	119	0.000***

***P < 0.001

## Discussion

With the advancement of high-throughput sequencing and bioinformatic techniques, the recognition and understanding of circRNAs has been widely extended. Research has confirmed that circRNAs play regulatory roles in PDAC progression [15, 33]. A previous study demonstrated that circFOKX2 not only interacts with YBX1 and hnRNPK to elevate the expression of the oncogenes NUF2 and PDXK but also functions as a sponge for miR-942 to upregulate the expression of ANK1, GDNF, and PAX6, which contribute to tumor progression in PDAC [25]. Another study demonstrated that hsa_circ_001653 facilitates the progression of PDAC through the miR-37/HOXC6 axis [34]. However, fewer studies have been conducted on circRNAs related to PDAC than on circRNAs related to other gastrointestinal tumors [15]. Through a series of experiments, our investigation was the first to report the existence of the circEYA3/miR-1294/c-Myc axis in the progression of PDAC (Fig. 8E).

In this study, we first identified differentially expressed circRNAs that were markedly upregulated in PDAC tissues compared with normal tissues in GEO datasets. Many circRNAs have low expression levels in both cancer cells and normal cells [35]. However, based on our preliminary screening of qRT-PCR results for clinical samples, we confirmed that circEYA3 was a circRNA markedly upregulated in PDAC tissues and that a higher expression level of circEYA3 was related to greater lymph node invasion and more advanced tumor stage. CircEYA3 was confirmed through Sanger sequencing to be derived from exons 2–6 of EYA3 by back-splicing, was found to be a highly stable circular transcript under treatment with RNase R and ActD, and was shown to be localized predominantly in the cytoplasm. Moreover, gain- and loss-of-function experiments demonstrated that circEYA3 acted as an oncogene to promote the proliferation, migration and invasion and inhibit the apoptosis of PDAC cells. Additionally, we found that circEYA3 performed its tumor-promoting functions by increasing ATP production in PDAC cells. Furthermore, FISH performed on our PDAC TMAs confirmed that high circEYA3 expression was significantly related to advanced tumor stage and poor patient OS, suggesting that circEYA3 may participate in the progression of PDAC.

CircRNAs play their regulatory roles by acting as miRNA sponges, encoding peptides, interacting with proteins, and regulating transcription or translation [7]. However, circRNAs mostly act as miRNA sponges in cancer cells, resulting in reductions in the expression and function of miRNAs [8, 35]. One of the well-known and best characterized circRNAs is ciRS-7 (CDR1as), which was shown to contain more than 60 binding sites for miR-7 and to obviously impair the activity of miR-7 in multiple cancer cells where it is highly expressed [36–39]. Given that circEYA3 is generated from the coding exons of EYA3 and localized predominantly in the cytoplasm, we hypothesized that circEYA3 may function as miRNA sponge to affect downstream target genes. Hence, we applied bioinformatic tools (circBank, starBase and miRanda) to predict the potential target of circEYA3 and initially identified 16 miRNAs as candidate targets of circEYA3. Notably, we performed a circEYA3 biotin pull-down assay and a RIP assay and confirmed that circEYA3 directly bound to miR-1294. Moreover, circEYA3 and miR-1294 were confirmed by a dual luciferase reporter assay and FISH to interact with each other and colocalize in the cytoplasm. Further statistical analysis indicated a negative correlation between circEYA3 and miR-1294 expression in PDAC tissues. Abnormal expression of miRNAs is proposed to be associated with proliferation and progression in different human cancers [40]. In fact, miR-1294 has been identified as a downregulated miRNA and a tumor suppressor in many types of cancer [27, 28], including PDAC [30]. Our results are consistent with those of previous studies and proved that miR-1294 is downregulated in both PDAC tissues and cells. Gain- and loss-of-function experiments showed that miR-1294 inhibited the proliferation and progression and decreased the ATP production of PDAC cells, suggesting that miR-1294 functioned as a tumor suppressor in PDAC. More importantly, further rescue experiments both in vivo and in vitro confirmed that miR-1294 reversed the oncogenic effects of circEYA3 in PDAC.

Previous studies have confirmed that miR-1294 inhibits the proliferation and migration of OSCC and ESCC cells by directly binding to the 3′ UTR of c-Myc [31, 32], a critical oncogene that drives pancreatic cancer tumorigenesis by controlling cell proliferation, metabolism, and apoptosis [16]. In the present study, c-Myc was confirmed to be a target of miR-1294 by a luciferase reporter assay in PDAC cells. We also found that miR-1294 overexpression suppressed the activation of c-Myc, indicating that miR-1294 was an important negative regulator of c-Myc in PDAC cells. Further statistical analysis showed a marked negative correlation between miR-1294 and c-Myc expression in PDAC tissues. Next, the western blot analysis results showed that altering the expression of circEYA3 greatly affected c-Myc expression both in vitro and in vivo and that miR-1294 reversed this change. The expression of circEYA3 was significantly positively correlated with that of c-Myc, based on our previous qRT-PCR data. Given the particular importance of c-Myc in PDAC progression [17, 22], our findings revealed that circEYA3 acted as a decoy to sequester miR-1294 in order to alleviate the suppression of its target gene c-Myc to in turn increase ATP production in PDAC cells, which can promote the progression of PDAC.

We first proved that circEYA3 promoted PDAC progression by inducing c-Myc expression by binding to miR-1294. However, our study has some limitations. First, not all circRNAs can function as miRNA sponges [41]. Whether circEYA3 regulates the proliferation and progression of PDAC cells via other biological processes, such as binding RBPs or being translated into a peptide, requires further investigation. Second, c-Myc is a well-known oncogenic transcription factor and can bind to E-box sequences in the promoters of numerous genes to regulate the expression of downstream genes [16, 42]. According to JASPAR (http://jaspar.genereg.net/), c-Myc shares binding sites with the promoter region of EYA3 (Fig. S5A). The silencing of c-Myc did not affect the level of EYA3 mRNA (Fig. S5B and S5C). Some transcription factors may affect the production of circRNAs [43, 44]. However, the silencing of c-Myc had no effect on the expression of circEYA3 (Fig. S5B). Thus, further studies are needed to explore the mechanism of upregulation of circEYA3 in PDAC. Our findings partially revealed that c-Myc can be regulated by circEYA3 in PDAC, although further in-depth research is needed to explore the more specific relationship between circEYA3 and c-Myc in PDAC. Third, our research preliminarily showed that circEYA3 was significantly elevated in PDAC tissues and cells. However, the expression of circEYA3 should be evaluated in a wider range of clinical samples, such as blood, urine, and saliva, to improve its diagnostic and therapeutic value.

## Conclusion

In conclusion, circEYA3 competitively binds miR-1294 to impair the suppressive effect of miR-1294 on c-Myc and increases energy production to facilitate the proliferation and progression of PDAC cells. Our findings expand the understanding of PDAC progression and shed light on therapeutic targets for PDAC.

## Supplementary Information

**Additional file 1.**

**Additional file 2.**

**Additional file 3.**

**Additional file 4.**

**Additional file 5.**

**Additional file 6.**

## Data Availability

All data that support the conclusion of this study are available from the corresponding authors on reasonable request.

## Abbreviations

PDAC: Pancreatic ductal adenocarcinoma
ATCC: American Type Culture Collection
EdU: 5-Ethynyl-2′-deoxyuridine
ADAR1: Adenosine deaminases acting on RNA 1
GLI1: Glioma-associated oncogene 1
CST: Cell Signaling Technology
EMT: Epithelial-to-mesenchymal transition
DMEM: Dulbecco’s modified Eagle’s medium
YBX1: Y-boxbinding protein
hnRNPK: Heterogeneous nuclear
PDXK: Pyridoxal kinase
NUF2: NUF2 component of NDC80 kinetochore complex.
ANK1: Ankyrin 1
GEO: Gene Expression Omnibus
OS: Overall survival
GDNF: Glial cell-derived neurotrophic factor
PAX6: Paired box 6
OSCC: Oral squamous cell carcinoma
ESCC: Esophageal squamous cell carcinoma
RBPs: RNA-binding proteins
PVDF: Polyvinylidene fluoride
BCA: Bicinchoninic acid
TMAs: Tissue microarrays
