# Supplementary material for: Circular RNA CircEYA3 induces energy production to promote pancreatic ductal adenocarcinoma progression through the miR-1294/c-Myc axis
Source: Mol Cancer. 2021 Aug 21;20:106. doi: 10.1186/s12943-021-01400-z (PMC8379744; doi:10.1186/s12943-021-01400-z)
Supplement: Supplementary file 1 — Additional file 1. [file 12943_2021_1400_MOESM1_ESM.docx]

**Additional file 1**

TableS1：The sequences of primers for qRT-PCR

| Sequence (5’-3’) | |
| --- | --- |
| circ[EYA3](http://www.ncbi.nlm.nih.gov/gene/?term=6533) forward | GACAGACTCAATACCAGACACTACAGC |
| circ[EYA3](http://www.ncbi.nlm.nih.gov/gene/?term=6533) reverse | TCTTGCTCTTCTTCCATGAGGACC |
| hsa_circ_0029634 forward | CCCTGTGCCTGTGTATATCCCAG |
| hsa_circ_0029634 reverse | ACAAGATTTGGTCTGCATGTGAGG |
| hsa_circ_0006117 forward | GACTCTGACAATGGGACCACAAG |
| hsa_circ_0006117 reverse | GAGCAGAACAAGAATGAACCAGG |
| hsa-miR-196b-5p forward | GCGCGTAGGTAGTTTCCTGTT |
| hsa-miR-196b-5p reverse | AGTGCAGGGTCCGAGGTATT |
| hsa-miR-196b-5p stem-loop | GTCGTATCCAGTGCAGGGTCCGAGGTATTCGCACTGGATACGACCCCAAC |
| hsa-let-7b-5p forward | GCGCGTGAGGTAGTAGGTTGT |
| hsa-let-7b-5p reverse | AGTGCAGGGTCCGAGGTATT |
| hsa-let-7b-5p stem-loop | GTCGTATCCAGTGCAGGGTCCGAGGTATTCGCACTGGATACGACAACCAC |
| hsa-let-7f-5p forward | CGCGCGTGAGGTAGTAGATTGT |
| hsa-let-7f-5p reverse | AGTGCAGGGTCCGAGGTATT |
| hsa-let-7f-5p stem-loop | GTCGTATCCAGTGCAGGGTCCGAGGTATTCGCACTGGATACGACAACTAT |
| hsa-miR-873-5p forward | CGCGGCAGGAACTTGTGAG |
| hsa-miR-873-5p reverse | AGTGCAGGGTCCGAGGTATT |
| hsa-miR-873-5p stem-loop | GTCGTATCCAGTGCAGGGTCCGAGGTATTCGCACTGGATACGACAGGAGA |
| hsa-let-7i-5p forward | CGCGCGTGAGGTAGTAGTTTGT |
| hsa-let-7i-5p reverse | AGTGCAGGGTCCGAGGTATT |
| hsa-let-7i-5p stem-loop | GTCGTATCCAGTGCAGGGTCCGAGGTATTCGCACTGGATACGACAACAGC |
| hsa-let-7a-5p forward | GCGCGTGAGGTAGTAGGTTGT |
| hsa-let-7a-5p reverse | AGTGCAGGGTCCGAGGTATT |
| hsa-let-7a-5p stem-loop | GTCGTATCCAGTGCAGGGTCCGAGGTATTCGCACTGGATACGACAACTAT |
| hsa-miR-3118 forward | CGCGTGTGACTGCATTATGAA |
| hsa-miR-3118 reverse | AGTGCAGGGTCCGAGGTATT |
| hsa-miR-3118 stem-loop | GTCGTATCCAGTGCAGGGTCCGAGGTATTCGCACTGGATACGACAGAATT |
| hsa-miR-1294 forward | GCGTGTGAGGTTGGCATTG |
| hsa-miR-1294 reverse | AGTGCAGGGTCCGAGGTATT |
| hsa-miR-1294 stem-loop | GTCGTATCCAGTGCAGGGTCCGAGGTATTCGCACTGGATACGACAGACAA |
| hsa-let-7c-5p forward | GCGCGTGAGGTAGTAGGTTGT |
| hsa-let-7c-5p reverse | AGTGCAGGGTCCGAGGTATT |
| hsa-let-7c-5p stem-loop | GTCGTATCCAGTGCAGGGTCCGAGGTATTCGCACTGGATACGACAACCAT |
| hsa-miR-196a-5p forward | CGCGCGTAGGTAGTTTCATGTT |
| hsa-miR-196a-5p reverse | AGTGCAGGGTCCGAGGTATT |
| hsa-miR-196a-5p stem-loop | GTCGTATCCAGTGCAGGGTCCGAGGTATTCGCACTGGATACGACCCCAAC |
| hsa-miR-98-5p forward | CGCGCGTGAGGTAGTAAGTTGT |
| hsa-miR-98-5p reverse | AGTGCAGGGTCCGAGGTATT |
| hsa-miR-98-5p stem-loop | GTCGTATCCAGTGCAGGGTCCGAGGTATTCGCACTGGATACGACAACAAT |
| hsa-miR-627-5p forward | GCGCGGTGAGTCTCTAAGAAA |
| hsa-miR-627-5p reverse | AGTGCAGGGTCCGAGGTATT |
| hsa-miR-627-5p stem-loop | GTCGTATCCAGTGCAGGGTCCGAGGTATTCGCACTGGATACGACTCCTCT |
| hsa-miR-296-3p forward | CGGAGGGTTGGGTGGAGG |
| hsa-miR-296-3p reverse | AGTGCAGGGTCCGAGGTATT |
| hsa-miR-296-3p stem-loop | GTCGTATCCAGTGCAGGGTCCGAGGTATTCGCACTGGATACGACGGAGAG |
| hsa-miR-134-5p forward | CGCGTGTGACTGGTTGACCA |
| hsa-miR-134-5p reverse | AGTGCAGGGTCCGAGGTATT |
| hsa-miR-134-5p stem-loop | GTCGTATCCAGTGCAGGGTCCGAGGTATTCGCACTGGATACGACCCCCTC |
| hsa-miR-380-3p forward | GCGCGTATGTAATATGGTCCA |
| hsa-miR-380-3p reverse | AGTGCAGGGTCCGAGGTATT |
| hsa-miR-380-3p stem-loop | GTCGTATCCAGTGCAGGGTCCGAGGTATTCGCACTGGATACGACAAGATG |
| hsa-miR-142-3p forward | GCGCGTGTAGTGTTTCCTACTT |
| hsa-miR-142-3p reverse | AGTGCAGGGTCCGAGGTATT |
| hsa-miR-142-3p stem-loop | GTCGTATCCAGTGCAGGGTCCGAGGTATTCGCACTGGATACGACTCCATA |
| [EYA3](http://www.ncbi.nlm.nih.gov/gene/?term=6533) forward | TGGTCAGAATCAGTACCAGGCC |
| [EYA3](http://www.ncbi.nlm.nih.gov/gene/?term=6533) reverse | GCCATGACACTAGGCTTCTCCG |
| c-Myc forward | GTGCTCCATGAGGAGACACCG |
| c-Myc reverse | CAGACTCTGACCTTTTGCCAGG |
| SURF4 forward | TGCAAATATTCCCCTGGTAGC |
| SURF4 reverse | GAAGAGAACCCATGTCCTGAAG |
| RNU6B stem-loop | CTCAACTGGTGTCGTGGAGTCGGCAATTCAGTTGAGAAAAATAT |
| RNU6B forward | TGCAGACGTGGCAAT |
| RNU6B reverse | TCAACTGGTGTCGTGG |
| GAPDH forward | GTCTCCTCTGACTTCAACAGCG |
| GAPDH reverse | ACCACCCTGTTGCTGTAGCCAA |

TableS2：The sequences to silence circ[EYA3](http://www.ncbi.nlm.nih.gov/gene/?term=6533) and c-Myc

| Sequence (5’-3’) | |
| --- | --- |
| si-circEYA3-1 | ACTACCTCCTTTTGATTGT |
| si-circEYA3-2 | ACCTCCTTTTGATTGTGAA |
| si-circEYA3-3 | TTTGATTGTGAAAACTGCT |
| si-c-Myc-nc | UUCUCCGAACGUGUCACGUTT |
| si-c-Myc-1 | CAGAAAUGUCCUGAGCAAUTT |
| si-c-Myc-2 | CGAGCUAAAACGGAGCUUUTT |

TableS3: Probes used in FISH.

|  | Sequence (5’-3’) |
| --- | --- |
| FISH Probes | |
| Cy3-circ[EYA3](http://www.ncbi.nlm.nih.gov/gene/?term=6533) | TTCACAATCAAAAGGAGGTAGTC |
| FAM-miR-1294 | AGACAACAATGCCAACCTCACA |

TableS4: Probes used in RNA Pull-down assay.

|  | Sequence (5’-3’) |
| --- | --- |
| Pull-down assay Probes | |
| Oligo probe | GACTAGACGAGTCCAGTAAA |
| circ[EYA3](http://www.ncbi.nlm.nih.gov/gene/?term=6533) probe | CAATCAAAAGGAGGTAGTCC |

TableS5: Correlation between circEYA3 and miR-1294 expression and clinicopathological parameters in PDAC (n=104)

| Characteristic | Total (104) | CircEYA3 expression  Low (33) High (71) | P value | miR-1294 expression  Low (75) High (29) | P value |
| --- | --- | --- | --- | --- | --- |
| Age  ≤60  > 60 | 54  50 | 20 34  13 37 | 0.227 | 36 18  39 11 | 0.198 |
| Sex  Male  Female | 52  52 | 20 32  13 39 | 0.140 | 39 13  36 16 | 0.512 |
| Tumor location  Head  Body/tail | 54  50 | 16 38  17 33 | 0.632 | 37 17  38 12 | 0.395 |
| Grade  High/Moderate  Low | 59  45 | 20 39  14 31 | 0.764 | 42 17  33 12 | 0.809 |
| Tumor stage  T1/T2  T3 | 79  25 | 24 55  9 16 | 0.599 | 57 22  18 7 | 0.998 |
| N stage  N0-1  N2 | 91  13 | 32 59  1 12 | 0.047* | 65 26  10 3 | 0.679 |
| TNM stage  I-IIA  IIB+III | 53  51 | 23 30  10 41 | 0.009** | 36 17  39 12 | 0.331 |
| Tumor size (cm)  ＜4  ≥4 | 78  26 | 24 54  9 17 | 0.651 | 58 20  17 9 | 0.377 |
| CA19-9 level  ≤37 U/mL  >37 U/mL | 18  86 | 6 12  27 59 | 0.872 | 13 5  62 24 | 0.991 |

*P<0.05, **P <0.01.
