# Supplementary material for: Circular RNA CircEYA3 induces energy production to promote pancreatic ductal adenocarcinoma progression through the miR-1294/c-Myc axis
Source: Mol Cancer. 2021 Aug 21;20:106. doi: 10.1186/s12943-021-01400-z (PMC8379744; doi:10.1186/s12943-021-01400-z)
Supplement: Supplementary file 2 — Additional file 2. [file 12943_2021_1400_MOESM2_ESM.docx]

**Additional file 2**

**Figure S1**

**
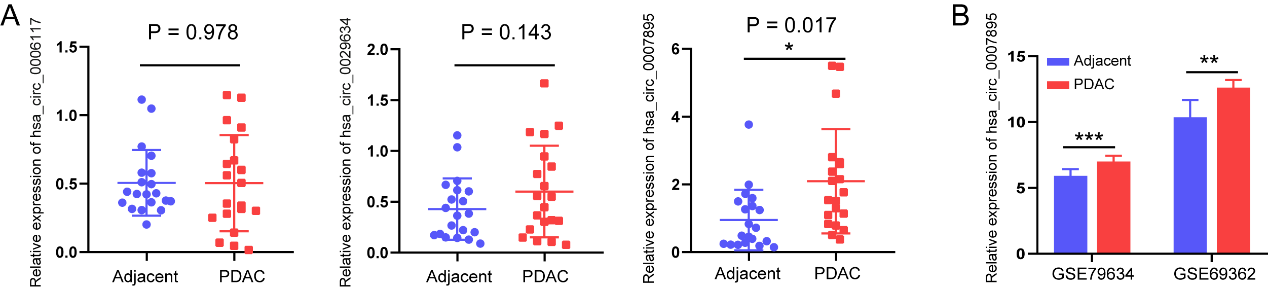
**

**Figure S1** **A.** The relative expression levels of hsa_circ_0029634, hsa_circ_0006117, and hsa_circ_0007895 in 20 pairs of PDAC and matched adjacent noncancerous tissues were evaluated by qRT-PCR. **B.** Relative expression levels of hsa_circ_0007895 in GSE79634 and GSE69362. *P < 0.05, **P < 0.01.
