# Supplementary material for: Circular RNA CircEYA3 induces energy production to promote pancreatic ductal adenocarcinoma progression through the miR-1294/c-Myc axis
Source: Mol Cancer. 2021 Aug 21;20:106. doi: 10.1186/s12943-021-01400-z (PMC8379744; doi:10.1186/s12943-021-01400-z)
Supplement: Supplementary file 3 — Additional file 3. [file 12943_2021_1400_MOESM3_ESM.docx]

**Additional file 3**

**Figure S2**

**
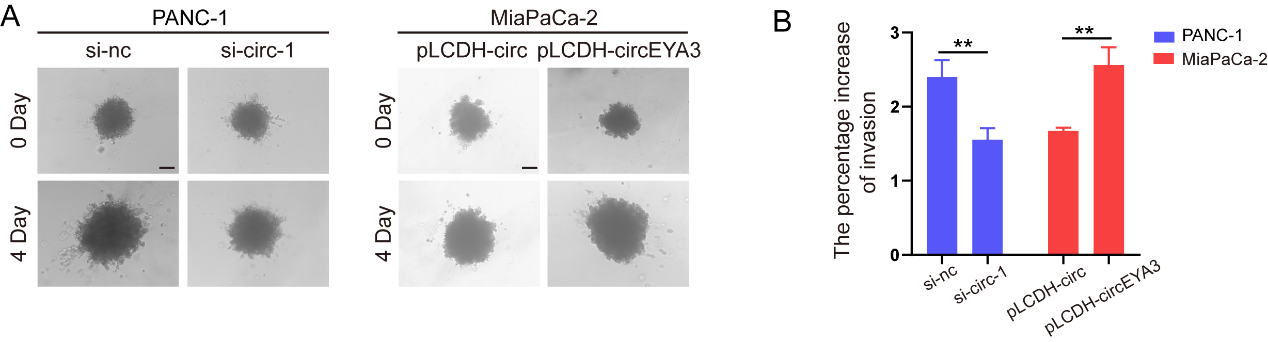
**

**Figure S2** **A and B.** 3D spheroid invasion assays were conducted to detect the invasive capabilities of PDAC cells. Scale bars, 100 μm. **P < 0.01.
