# Supplementary material for: Circular RNA CircEYA3 induces energy production to promote pancreatic ductal adenocarcinoma progression through the miR-1294/c-Myc axis
Source: Mol Cancer. 2021 Aug 21;20:106. doi: 10.1186/s12943-021-01400-z (PMC8379744; doi:10.1186/s12943-021-01400-z)
Supplement: Supplementary file 4 — Additional file 4. [file 12943_2021_1400_MOESM4_ESM.docx]

**Additional file 4**

**Figure S3**

**
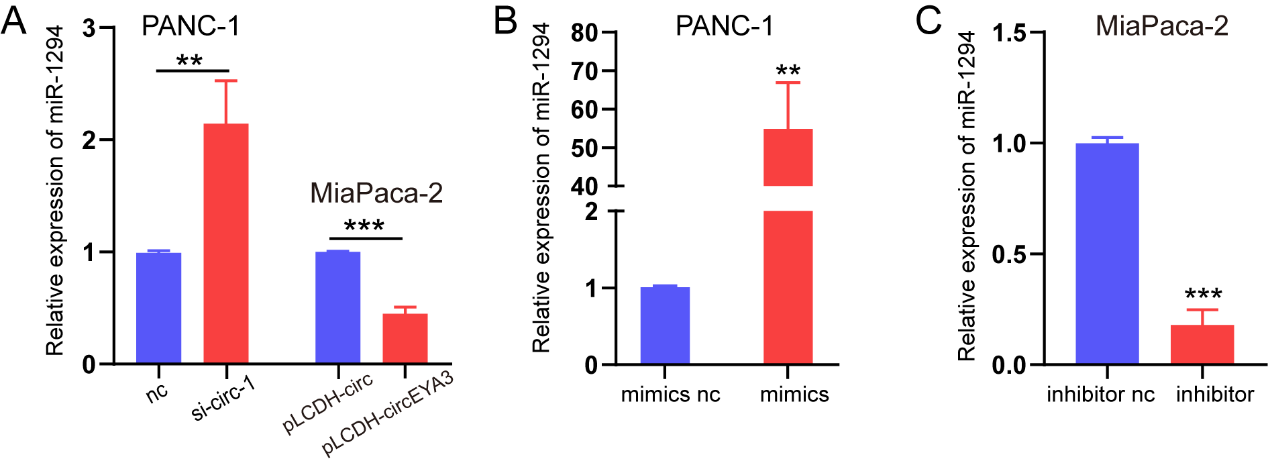
**

**Figure S3** **A.** Effects of the alteration of circEYA3 expression on miR-1294 expression. **B and C.** The efficiency of the miR-1294 mimic (**B**) and inhibitor (**C**) was evaluated by qRT-PCR in PDAC cells. **P < 0.01, ***P < 0.001.
