# Supplementary material for: Circular RNA CircEYA3 induces energy production to promote pancreatic ductal adenocarcinoma progression through the miR-1294/c-Myc axis
Source: Mol Cancer. 2021 Aug 21;20:106. doi: 10.1186/s12943-021-01400-z (PMC8379744; doi:10.1186/s12943-021-01400-z)
Supplement: Supplementary file 5 — Additional file 5. [file 12943_2021_1400_MOESM5_ESM.docx]

**Additional file 5**

**Figure S4**

**
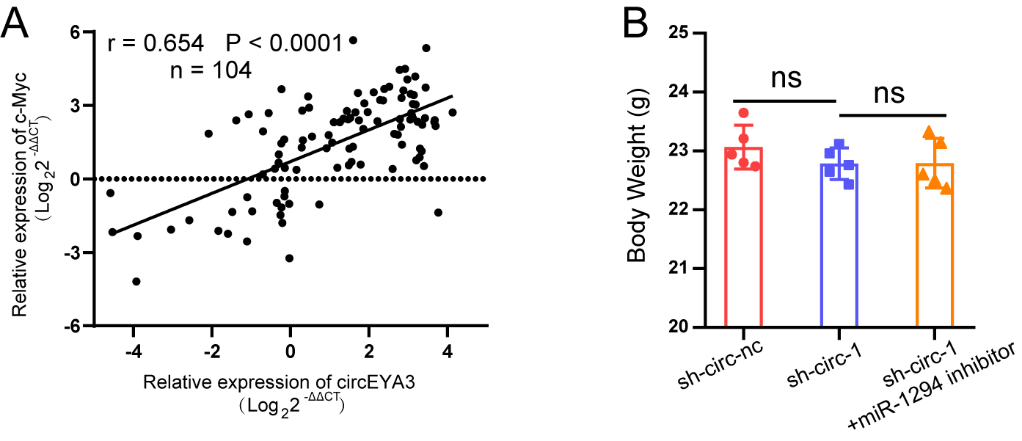
**

**Figure S4 A.** Correlation between circEYA3 and miR-1294 expression in PDAC tissues, as determined by Pearson correlation analysis (n=104). **B.** The final body weights were evaluated in three groups. ns indicates no significance.
