# Supplementary material for: Circular RNA CircEYA3 induces energy production to promote pancreatic ductal adenocarcinoma progression through the miR-1294/c-Myc axis
Source: Mol Cancer. 2021 Aug 21;20:106. doi: 10.1186/s12943-021-01400-z (PMC8379744; doi:10.1186/s12943-021-01400-z)
Supplement: Supplementary file 6 — Additional file 6. [file 12943_2021_1400_MOESM6_ESM.docx]

**Additional file 6**

**Figure S5**

**
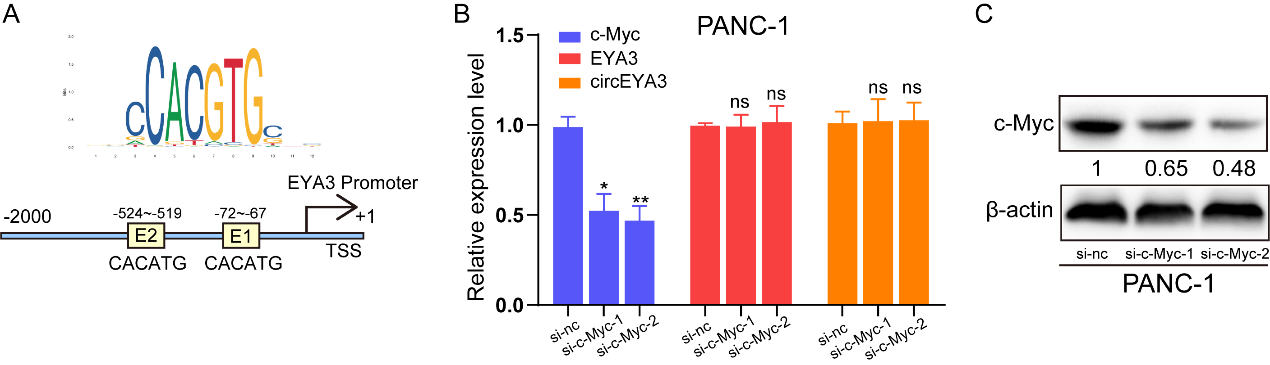
**

**Figure S5** **A.** Schematic illustration of the [potential](javascript:;) c-Myc binding sites in the promoter region of EYA3. **B.** The relative expression levels of circEYA3 and EYA3 were evaluated by qRT-PCR after silencing of c-Myc in PANC-1 cells. **C.** The effect of silencing c-Myc was evaluated by western blot analysis. *P < 0.05, **P < 0.01; ns indicates no significance.
